# Investigations on interpolymer complexes of cationic guar gum and xanthan gum for formulation of bioadhesive films

**Published:** 2010

**Authors:** M. Singh, A.K. Tiwary, G. Kaur

**Affiliations:** 1 *Ranbaxy Research Labs, Plot No. 20, Sector 18, Gurgaon, India*; 2 Department of Pharmaceutical Sciences and Drug Research, Punjabi University, Patiala-147002, India

**Keywords:** Bioadhesion, Cationic guar gum, Xanthan gum, Interpolymer complexation, Buccal film

## Abstract

The present study was aimed at evaluating the possible use of inter polymer complexed (IPC) films of xanthan gum (XG) and cationic guar gum (CGG) for formulating domperidone bioadhesive films. Formation of bonds between –COO¯ groups of XG and –N^+^(CH_3_)_3_ groups of CGG was evident in the FTIR spectra of IPC films. Bioadhesive strength of the films was evaluated employing texture analyser. Water uptake studies indicated swelling to be a function of XG concentration in the interpolymer complexes. The bioadhesive films were found to possess neutral pH. *In vitro* drug release studies and residence time studies indicated that the film comprising CGG:XG (80:20) released 98% of domperidone in 8 h and exhibited a residence time of approximately 8 h. Enhanced bioavailability of domperidone was observed from bioadhesive films as compared to orally administered conventional tablets. Overall, the findings suggest that IPC films of XG and CGG, exhibiting desired bioadhesive strength and enhanced bioavailability of domperidone, can be prepared.

## INTRODUCTION

The focus of pharmaceutical research is being steadily shifted from the development of new chemical entities to the development of novel drug delivery system of existing drug molecules to maximize their effectiveness in terms of therapeutic action and patient protection. Conventional drug delivery, in most cases, gives poor control of plasma drug concentration. Constant plasma drug levels can be attained by designing a dosage form with ability to release its contents at a continuous and controlled rate for a long duration of time. Buccal adhesive systems offer innumerable advantages in terms of accessibility, administration and withdrawal, low enzymatic activity, economy and high patient compliance(1).

Cationic guar gum (CGG) is modified guar gum in which hydroxyl groups are replaced with trimethyl ammonium groups. The introduction of trimethyl ammonium groups imparts cationic character to the gum. It acts as excellent non-gelling thickener, viscosity, volume and foam enhancer. Due to ammonium groups, it carries a net positive charge and can be easily cross-linked with other anions. Xanthan gum (XG) is a polysaccharide having polyanionic properties due to carboxylic groups and has very good bioadhesive strength. However, despite their biodegradable character neither CGG nor XG can be used alone to formulate a buccoadhesive formulation as both possess highly acidic or alkaline pH due to the presence of anionic or cationic groups, respectively. The extreme acidic or alkaline pH, gives rise to mucosal irritation(2). The presence of a number of amino groups permits CGG to chemically react with XG, thereby resulting in alteration of physiochemical characteristics and release behavior of such combinations(3).

Domperidone is a peripheral dopamine antagonist with antiemetic and gastroprokinetic properties(4). It increases esophageal peristalsis and lowers esophageal sphincter pressure, increases gastric motility and peristalsis, and consequently facilitates gastric emptying and decreases small bowel time. However, it undergoes extensive gut wall metabolism and first pass metabolism in liver, resulting in an absolute bioavailability of approximately 15%. Since the drugs administered by buccoadhesive systems bypass the first pass metabolism, the bioavailability of domperidone can be envisaged to increase by administering it from buccoadhesive films. Further, buccoadhesive films can be envisaged to ensure maintenance of effective plasma concentration over prolonged duration by extending the release of domperidone. This is expected to reduce the frequency of administration by maintaining effective plasma concentration over prolonged duration.

In the light of above facts, it was proposed to formulate bioadhesive films of domperidone comprising of interpolymer complex (IPC) of CGG and XG. The characterization of the IPC films was done by FTIR studies. The effect of varying the composition of XG and CGG in the IPC films on the bioadhesive strength and drug release was evaluated. Further, the formulated domperidone films were evaluated for their pharmacokinetic performance after oral administration to the rabbits.

## MATERIALS AND METHODS

Domperidone was received as a gift sample from Nayan Pharmaceuticals (Patiala, India). Cationic guar gum was received as gift sample from Encore Natural Polymers Pvt. Ltd. (Ahmadabad, India). Xanthan gum was received as gift sample from Panacea Biotech Ltd. (Lalru, Chandigarh, India). Ammonium acetate and acetic acid were of analytical grade and purchased from Qualigens Fine Chemicals (India). Acetonitrile, potassium dihydrogen orthophosphate and methanol of HPLC grade were purchased from Merck India, Ltd. All reagents and chemicals were of analytical grade and used as received.

### Stoichiometry of the CGG and XG polymer complex

CGG solution (2% w/v) was prepared in 2% v/v acetic acid. XG solution (2% w/v) was separately prepared by hydrating in distilled water. Equal volumes of CGG and XG solutions were mixed at 25 °C. The samples were incubated at 37 °C for 24 h. The samples were then centrifuged at 15000 rpm. The viscosity of the supernatant solution was determined using Brookefield RVDV II Pro Viscometer, UK (Spindle 21).

### Preparation of drug loaded and unloaded buccoadhesive films comprising IPC films between CGG and XG

XG (20 mg) was dissolved in 40 ml distilled water. CGG (80 mg) was separately dissolved in 40 ml solution of 2% v/v acetic acid by magnetic stirring for 1 h. Ammonium acetate solution (5 M, 10 ml) was added to both the above solutions. XG solution was slowly added with continuous stirring to CGG solution. This mixture was poured in petriplates and dried at 50°C for 48 h. Films with a total polymer content of 2% w/v containing 80:20 (C1), 70:30 (C2), 60:40 (C3), 50:50 (C4), 40:60 (C5), 30:70 (C6) or 20:80 (C7) ratio of CGG:XG were prepared using this method. The dried films were carefully removed, checked for any imperfections or air bubbles and stored in a desiccator until use. Similarly drug loaded buccoadhesive films were prepared by dissolving domperidone (185 mg) in CGG solution. The final mixture containing drug, CGG and XG was poured in petriplates and dried at 50 °C for 48 h.

### Characterization of IPC films

#### Swelling index measurement

The swelling index of the IPC films was determined by immersing the films in 5.0 ml phosphate buffer pH 6.6 and removing them at time intervals of 0.5, 1, 2, 3, 4, 5, 6 or 7 h. The excess water on the surface of the film was carefully removed using tissue paper and the swelling index was calculated using the following formula

$$Swelling\;index\;=\;\frac{W2\;-\;W1}{W1}$$

where, W1 is the initial weight of the film and W2 is the weight of the swollen film. The procedure was repeated six times.

#### FTIR analysis

XG, CGG powder and the IPC films formed by drying admixtures containing different ratios of XG:CGG were subjected to FTIR analysis (Perkin Elmer RXI, USA).

### Evaluation of physical parameters of the films

The formulated films were tested for mass uniformity, thickness and folding endurance. Mass uniformity was tested in 10 different randomly selected films from each batch and film thickness was measured at 5 different randomly selected spots using a vernier caliper. Folding endurance of the films was determined by repeatedly folding one film at the same place till it broke or folded up to 200 times without breaking(5).

### Surface pH determination

The surface pH was determined by the method reported by Bottenberg et al.(6). A combined glass electrode was used for this purpose. The films were allowed to swell by keeping them in contact with buffer (pH 6.5 ± 0.1) for 2 h at room temperature. The pH was noted down by bringing the electrode in contact with the surface of the film, allowing it to equilibrate for 1 min.

### Bioadhesive strength

Porcine cheek mucosa was used as a model membrane and acetate buffer (pH 6.0) as moistening fluid for measurement of bioadhesive strength. The surface of the mucosal membrane was first blotted with a filter paper and then moistened with phosphate buffer pH 6.6. The porcine mucosa (thickness 0.05 ± 0.01 mm) was attached with double sided adhesive tape to the lower stationary part of the TA-XT Plus Texture Analyzer (Stable Microsystems, UK). A piece of free film to be tested (thickness 0.5 ± 0.01 mm) was attached to the upper movable part. The upper part was lowered until it reached in contact with the porcine mucosa. A preload of 200 g(7) was applied for 100 s, after which the upper part was raised with a speed of 0.01 mm/s. Each experiment was performed using porcine cheek mucosa obtained from five different animals, and force of detachment was recorded.

### In vitro drug release studies

The *in vitro* dissolution studies were carried out using the USP 28 type 5-paddle(8) method using 900 ml of phosphate buffer (pH 6.6) as the dissolution medium. The studies were carried out at 50 rpm at 37 ± 0.5 °C for 8 h. To provide unidirectional release, one side of each film was attached on glass disk with the help of cyanoacrylate instant adhesive(9). An aliquot of 5 ml sample was withdrawn at suitable time intervals and similar volume was replaced with fresh phosphate buffer (pH 6.6) maintained at the same temperature. The samples were then analyzed spectrofluorometrically employing excitation wavelength of 286 nm and emission wavelength at 328 nm. Each experiment was carried out in triplicate.

### Ex vivo mucoadhesion time

The selected batch was subjected to ex vivo mucoadhesion test. The disintegration medium composed of 800 ml isotonic phosphate buffer pH 6.6 (IPB) maintained at 37 °C. A segment of porcine cheek mucosa, 3 cm long, was glued to the surface of a glass slab, vertically attached to the apparatus. The mucoadhesive film was hydrated from one surface using 15 μl IPB and then the hydrated surface was brought into contact with the mucosal membrane. The glass slab was vertically fixed to the apparatus and allowed to move up and down so that the film was completely immersed in the buffer solution at the lowest point and was out at the highest point. The time necessary for complete erosion or detachment of the film from the mucosal surface was recorded. The experiment was carried out in triplicate(10).

### In situ release studies

The studies were carried out by using Keshary-Chein glass diffusion cells. The porcine cheek pouch was pretreated by attaching it between the receptor and donor compartment with the help of clamp. The whole assembly was maintained at 37 ± 1°C, and the medium was stirred at 100 rpm. An aliquot of sample (1 ml) was taken at suitable time intervals from the receptor compartment and equal volume was replaced with fresh phosphate buffer (pH 7.4) maintained at the same temperature. The samples were analyzed spectrofluorimetrically as for dissolution samples.

### Pharmacokinetic studies

Pharmacokinetic studies were carried out in rabbits. The buccal membrane of rabbits closely resembles human buccal membrane in terms of structure and permeability(11). Rabbits weighing 3-4 kg were housed in separate cages. The animals selected for the study had no medication for two weeks prior to the study. They were restrained from eating and drinking during the study with buccal film. The formulated test film was placed in the buccal position of oral cavity with polymer side facing the mucosa of buccal cavity. Ethylcellulose membrane was stuck to the other side of film using cyanoacrylate glue to prevent drug release. A gentle pressure was applied for one min. The rabbits were sedated with an intramuscular injection of ketamine (25 mg/kg) before application of test film. Blood samples were taken from ear vein after regular intervals for 8 h, and collected in 1.5 ml micro centrifuge tubes containing 40 μl of heparin and then centrifuged at 4000 rpm for 10 min to separate plasma. The separated plasma was stored at -20 °C until analyzed. The bioavailability from buccal film containing approximately 7 mg of domperidone was compared with that of oral solution containing 10 mg of domperidone in phosphate buffer containing 20% v/v of absolute ethanol.

## RESULTS

**Fig. 1:**
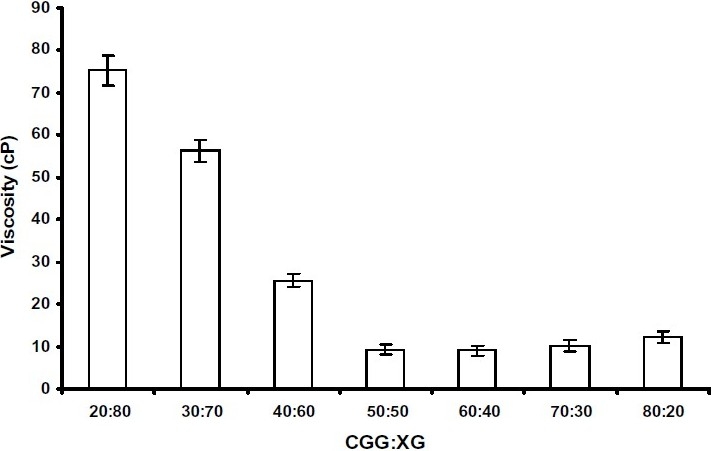
Viscosity of the supernatant obtained after centrifugation of mixtures containing varying ratios of CGG and XG.

**Fig. 2:**
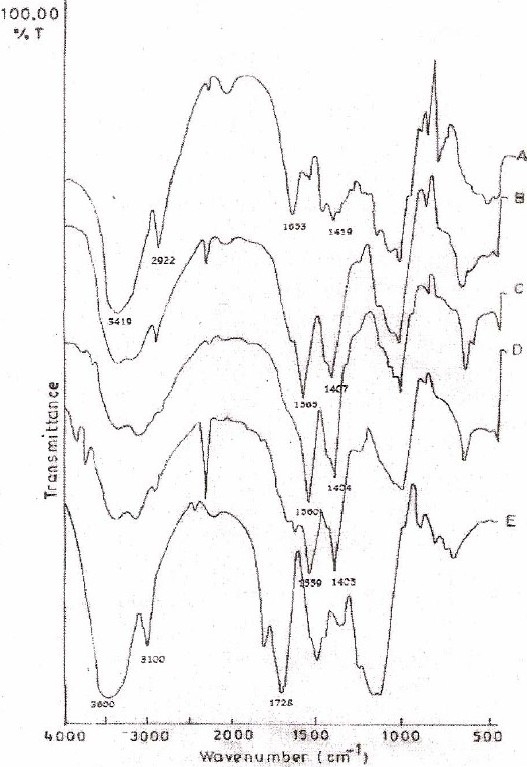
FTIR spectra of CGG (A), XG (E) and IPC film comprising of 80:20 (B), 50:50 (C) or 20:80 (D)CGG:XG ratios.

Fig. 1 depicts the viscosity of the supernatant obtained after centrifugation of mixtures containing varying ratios of aqueous solutions of XG and CGG. Fig. 2 depicts the FTIR spectra of CGG, XG and various IPC films of CGG and XG. Sharp peaks of strong intensity were observed at 1653 cm^-1^ and at 1419 cm^-1^ in the FTIR spectra of CGG. The FTIR spectra of XG showed peaks at 1728 cm ^-^ 1 whereas the IPC films exhibited peaks at 1565 cm^-1^ and 1407 cm^-1^.

**Fig. 3:**
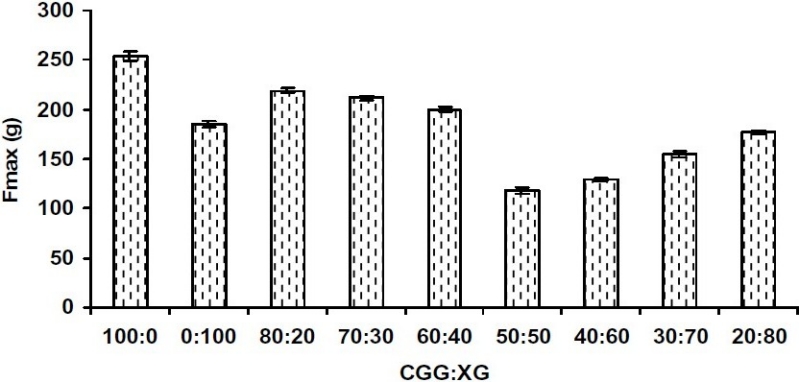
Force required for detachment of bioadhesive film from pig cheek mucosa.

Fig. 3 shows the peak force obtained from *in vitro* tensile tests for the different IPC films. The data of physical parameters like mass, thickness, drug content (%), folding endurance of all the formulations is summarized in Table 1. Swelling index of all investigational formulations (batch A, B, C1, C2, C3, C4, C5, C6, C7) observed at 8.0 h in phosphate buffer pH 6.6 and surface pH is shown in Table 1.

**Fig. 4:**
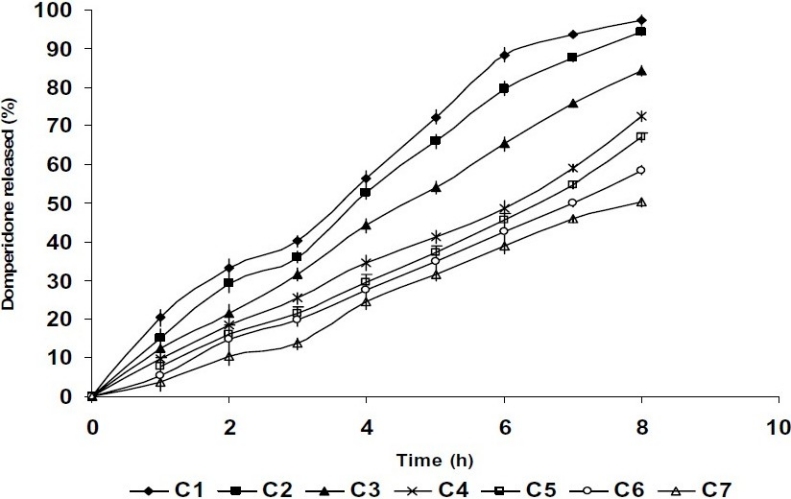
*in vitro* drug release studies from formulated bioadhesive films.

Fig. 4 shows the *in vitro* release profile of bioadhesive films. Batch C1 and C 2 released 97.29% and 94.45% of domperidone, respectively in 8 h. Batch C1 comprising 80:20 ratio of CGG:XG exhibited residence time of 415 ± 15 min. *In situ* diffusion studies carried out on batch C1 and C2 showed release of 91.89% and 85.54% of domperidone, respectively in 8 h.

The plasma concentration of domperidone after application of batch C1 to rabbits gradually increased and attained maximum in 4 h. The pharmacokinetic parameters such as C_max_, T_max_, AUC_total_ are summarized in Table 2. The pharmacokinetic parameters of domperidone after application of buccal film were significantly different (*P*<0.05) from that obtained after oral administration of the tablet.

**Table 1:** Buccoadhesive indices of formulated bioadhesive films

Batch code		IPC ratio (CGG:XG)	Domperidone (mg)	Mass (mg)	Folding endurance	Drug content (%)	Swelling index (after 8 h)	*Ex vivo* mucoadhesion time (min)
A		100:0	185	198 ± 1	195 ± 2	97.84 ±	3.57 ± 0.05	ND*
B		0:100	185	202 ± 3	200 ± 3	98.45 ±	7.34 ± 0.03	ND*
	C1	80:20	185	196 ± 2	198 ± 2	98.66 ±	4.37 ± 0.04	415 ± 15
	C2	70:30	185	192 ± 7	196 ± 4	99.21 ±	5.12 ± 0.02	387 ± 22
	C3	60:40	185	204 ± 3	199 ± 4	99.36 ±	5.48 ± 0.05	312 ± 16
	C4	50:50	185	198 ± 2	198 ± 3	98.75 ±	5.88 ± 0.02	254 ± 10
C	C5	40:60	185	194 ± 3	198 ± 3	100.2 ±	6.17 ± 0.05	287 ± 18
	C6	30:70	185	195 ± 4	200 ± 4	97.52 ±	6.49 ± 0.04	328 ± 21
	C7	20:80	185	190 ± 7	196 ± 3	100.8 ±	6.67 ± 0.03	345 ± 142

ND: not determined

* Since the pH of the films containing CGG or XG alone was too basic or acidic for application on buccal mucosa, herefore further studies were not carried out on these films.

**Table 2:** Pharmacokinetic parameters of formulated bioadhesive film comprising 80:20 (CGG:XG)

Parameter	C_max_ (ng/ml	AUC_total_ (ng.h/ml)
Buccal Film	2287 ± 114	6706 ± 315.8
Tablet	1393 ± 25.7	3936 ± 234.6

## DISCUSSION

CGG bears a net positive charge due to presence of –N^+^ (CH_3_)_3_ groups, whereas XG is negatively charged due to the presence of – COO¯ groups. As a result, when these polymers are mixed together, they form a solid mass. In the present study, the stoichiometric ratio of the IPC formed was confirmed by employing viscosity measurements. It was observed that there was a sharp decrease in viscosity when the proportion of XG in the CGG-XG was decreased (Fig. 1). Similar studies by Kaur et al. revealed that the viscosity of the supernatant obtained after mixing aqueous solutions of chitosan and carboxymethyltamarind kernel powder decreases with a decrease in the proportion of chitosan in these mixtures(12). The minimum viscosity of the supernatant observed at CGG:XG ratio of 50:50 indicated maximum interaction.

The swelling behavior of formulation governs its bioadhesion and drug release pattern. Swelling of the matrix, as indicated by a transition of the polymer from glassy to rubbery state, is an important parameter in the determination of release characteristics of the matrix system. The prepared films were cut and placed in phosphate buffer pH 6.6 at 37 ± 0.5 °C to simulate buccal conditions. Batch B (100% XG) showed highest swelling index within 1 h due to greatest swelling of XG (~700% weight increase in 8 h), indicating high water uptake and a small degree of erosion due to polymer relaxation(13). Batch A (100% CGG) exhibited lowest swelling index (~300% weight increase in 8 h). The results of the swelling studies (Table 1) indicated that the rate of swelling was a function of XG content and was inversely proportional to the CCG content of the films. XG, being a hydrophilic anionic polymer, has much higher affinity for water(14,15). When the film is placed in an aqueous medium, liquid penetrates into the film and a gel is formed due to uncoiling of the structure of XG molecules and formation of hydrogen bonds with water molecules. As a result, the diameter of the film increases progressively and a distinct gel-sol boundary develops. Thus the overall dimensions of the film are affected and rate of swelling is increased.

IR spectra of CCG (Fig. 2, A) showed a sharp peak of strong intensity at 1653 cm^-1^ and 1419 cm^-1^ which is the characteristic peak of amino group in CCG. XG (Fig. 2, E) exhibited sharp peak at 1728 cm^-1^ indicating the presence of carboxyl groups.

IR spectra of IPC films showed sharp peaks of strong intensity at 1565 cm^-1^ and 1407 cm^-1^ for CCG:XG (80:20), 1560 cm^-1^ and 1404 cm^-1^ for CCG:XG (50:50) and 1559 cm^-1^ and 1405 cm^-^ 1 for CCG:XG (20:80) (Fig. 2, B, C and D). The peak at 1560 cm^-1^ in the FTIR spectra of IPC films in (Fig. 2B, C and D could be attributed to –N^+^ (CH_3_)_3_ of CGG. The peak evident at 1407 cm^-1^ indicated the presence of –COO¯ ions (representing C=O stretch of – COO¯). These results indicate that the carboxylic groups of XG are dissociated into – COO¯ ions which complex with cationic groups of CGG through electrostatic interaction to form the poly-electrolyte complexes. Hence, the presence of –COO¯ groups and –N^+^ (CH_3_)_3_ in the IPC films strongly suggests the existence of N(CH_3_)_3_^+^COO¯ complex between CGG and XG molecule thus, leading to interpolymer complexation.

The films formulated using CGG alone (Batch A) showed very good bioadhesive strength of 254 g (Fig. 3). This strong mucoadhesive property of CGG could be due to the formation of ionic bonds between the positively charged (–N+(CH_3_)_3_) groups of CGG and the negatively charged sialic acid residues of mucin glycoproteins(16). The batch C1 comprising 80:20 ratio of CGG and XG exhibited higher bioadhesive strength as compared to all other ratios of CGG and XG. This observation can be explained by the presence of CGG in the cationic (protonated) form in the polymer complex. When CGG is in cationic form, electrostatic interaction with negatively charged mucus is possible and by this mechanism, CCG produces mucoadhesion(16). A decrease in CGG concentration in batches C2, C3, C4 led to a decrease in the force required to detach the films from mucosal membrane. Viscosity analysis of the supernatant obtained after mixing different ratios suggested maximum interaction at 50:50 ratios of CGG-XG. The positively charged groups of CGG formed ionic complex with the –COO– groups of XG, thus leading to the neutralization of the charge presented on both the polymers. It is already reported that polymers with neutral charge exhibit lesser mucoadhesive strength as compared to charged polymers, thus leading to decreased bioadhesive strength.(17). However, there was an increase in the force required for detachment of films of C5, C6 and C7 batches comprising 40:60, 30:70 or 20:80 ratio of CGG:XG, respectively. This could be due to the presence of free –COO¯ groups of XG. Physical entanglements and secondary interactions (hydrogen bonds) contribute to the formation of a strengthened network; therefore polymers that exhibit a high density of available hydrogen bonding groups (–COO® groups of XG) are able to interact more strongly with mucin glycoproteins(18).

The surface pH of the formulations (C1, C2, C3, C4, C5, C6, C7) was found to be within + 1.0 units of the neutral pH (Table 1). Therefore, these formulations can be expected to be non-irritating to the buccal mucosa. The surface pH of batch A (CGG:XG; 100:0) and B (CGG:XG; 0:100) was found to be 8.3 and 3.5, respectively. The pH of mucosa is reported to be 6.8(19). Therefore batches containing CGG or XG alone, although showed good bioadhesive properties, cannot be used for formulation of buccoadhesive films since the extreme pH values associated with them could produce irritation in the buccal mucosa.

*In vitro* drug release studies from batch A or B containing CGG alone or XG alone respectively, were not performed since the surface pH of these batches was found to be 8.3 and 3.5. The *in vitro* drug release profile of bioadhesive films is depicted in Fig. 4. An inverse relationship was found to exist between swelling index and drug release of bioadhesive films comprising different ratios of CGG and XG. The batches containing 80:20 and 70:30 ratio of CGG:XG released 97.29% or 94.45% of domperidone, respectively, whereas in all other batches less than 85% of domperidone was released till 8 h. It has already been reported that the release of a soluble drug like domperidone from hydrophilic matrices e.g., XG, proceeds through the gel layer (boundary layer control) which is formed surrounding the film upon contact with the medium. As the gel thickness increases, the diffusion path length increases, which in turn causes a decrease in drug release from the matrices(14). Analysis of the release data using Korsmeyer-Peppas kinetic model demonstrated that the drug was following Non Fickian release mechanism thus indicated that the drug release mechanism may involve a combination of both diffusion and chain relaxation mechanism. Therefore, the release of the drug from the formulated films is controlled by swelling of the polymer, followed by drug diffusion through the swelled polymer and slow erosion of the polymer(20).

The buccal administration of domperidone films (C1) resulted in maximum plasma concentration (C_max_) of 2287 ± 114.2 ng/ml after 4 h whereas, the oral administration of tablet produced significantly lower C_max_ of 1393 ± 25.7 ng/ml in 1 h. The AUC_total_ of domperidone with buccal film was found to be significantly higher as compared to conventional tablets. Domperidone has been reported to possess low bioavailability (15%) due to extensive first pass metabolism. Adhesion of buccal adhesive drug delivery devices to mucosal membranes leads to an increased drug concentration gradient at the absorption site and therefore improved bioavailability of systemically delivered drugs. The buccal formulation (C1) selected for *in vivo* study was found to enhance the bioavailability of domperidone by 1.7 times with reference to a 10 mg oral tablet of domperidone (Table 2).

## CONCLUSION

The results of the present study indicate that interpolymer complex between CGG and XG can be used to formulate bioadhesive films of domperidone. The results of bioadhesive strength studies demonstrated good interaction between the IPC and mucin on the mucus membrane of porcine cheek. The dissolution studies indicated that batch C1, comprising of 80:20 (CGG:XG), was able to release 95% of drug within a period of 8 h and exhibited a residence time of approximately 8 h. Further, the bioadhesive batch was found to be 1.7 times more bioavailable as compared to the marketed 10 mg oral tablet of domperidone.

## References

[1] Salamat-Miller N, Chittichang M, Johnston TP (2005). The use of buccoadhesive polymers in drug delivery. Adv Drug Del Rev.

[2] Fathia C, Maryam T, Severian D, Esteban ID (2000). Study of biodegradation behaviour of chitosanxanthan microspheres in simulated physiological media. J Biomed Mater.

[3] Singla AK, Chawla M, Singh A (2002). Potential applications of carbomer in oral mucoadhesive controlled drug delivery system: a review. Drug Dev Ind Pharm.

[4] Nagarsenker MS, Garad SD, Ramparkash G (2000). Design, optimization and evaluation of domperidone coevaporates. J Control Rel.

[5] Khurana R, Ahuja A, Khar RK (2000). Development and evaluation of mucoadhesive films of miconazole nitrate. Ind J Pharm Sci.

[6] Bottenberg P, Cleymact R, Muynck CD, Remon JP, Coomans D, Michotte Y (1991). Development and testing of bioadhesive, fluoride containing slow release tablets for oral use. J Pharm Pharmacol.

[7] Hagesaether E, Hiroth M, Sande SA (2009). Mucoadhesion and drug permeability of free mixed films of pectin and chitosan: an *in vitro* and *ex vivo* study. Eur J Pharm Biopharm.

[8] (2007). The United States Pharmacopoeia, 30th rev, and The National Formulary.

[9] Desai KGH, Kumar TMP (2004). Preparation and evaluation of a novel buccal adhesive systems. AAPS Pharm Sci Tech.

[10] Nafee NA, Ismail FA, Boraie NA, Mortanda LM (2003). Design and characterization of mucoadhesive buccal films containing cetylpyridium chloride. Acta Pharm.

[11] Dowty ME, Knuth KE, Robinson JR (1992). Enzyme characterization studies on the rate limiting barrier in the rabbit buccal mucosa. Int J Pharm.

[12] Kaur G, Jain S, Tiwary AK (2010). Chitosancarboxymethyl tamarind kernel powder interpolymer complexation: investigations for colon drug delivery. Sci Pharm.

[13] Mundargi RC, Patil SA, Aminabhavi M (2007). Evaluation of acrylamide-grafted-xanthan gum copolymer matrix tablets of oral controlled delivery of antihypertensive drugs. Carbohydrate Poly.

[14] Talukdar MM, Kinget R (1995). Swelling and drugrelease behaviour of xanthan gum matrix tablets. Int J Pharm.

[15] Huang Y, Huiqun Y, Xiao C (2007). pH sensitive cationic guar gum/poly(acrylic acid) polyelectrolyte hydrogels: swelling and *in vitro* drug release. Carbohydrate Poly.

[16] Lehr CM, Bouwastra JA, Schacht EH, Junginger HE (1992). *in vitro* evaluation of mucoadhesive properties of chitosan and some other polymers. Int J Pharm.

[17] Asane GS, Nirmal SA, Rasal KB, Naik AA, Mahadik MS, Rao YM (2008). Polymers for mucoadhesive drug delivery system: current status. Drug Dev Ind Pharm.

[18] Andrrews GP, Laverty TP, Jones DS (2009). Mucoadhesive polymeric platforms for controlled drug delivery. Eur J Pharm Biopharm.

[19] Aframian DJ, Davidowitz T, Benoliel R (2006). The distribution of oral mucosal pH values in healthy saliva secretors. Oral Dis.

[20] Mohammed FA, Kehdr H (2003). Preparation and *in vitro* evaluation of the buccal bioadhesive properties of slow release tablets containing miconazole nitrate. Drug Dev Ind Pharm.

